# The Clinical Value of the Ferning Test in Monitoring Dry Eye Syndrome in Patients with Sarcoidosis

**DOI:** 10.3390/life15091464

**Published:** 2025-09-18

**Authors:** Călina Anda Sandu, Cosmin Victor Ganea, Vlad Constantin Donica, Anisia Iuliana Alexa, Ioana Alexandra Sandu, Madalina Ioana Bilha, Camelia Margareta Bogdănici

**Affiliations:** 1 Ophthalmology Department, Grigore T. Popa University of Medicine and Pharmacy, 700115 Iasi, Romania; calina-anda.sandu@umfiasi.ro (C.A.S.); vlad-constantin.donica@umfiasi.ro (V.C.D.);; 2 Arcadia Medical Rehabilitation Hospital, 707035 Barnova, Romania

**Keywords:** dry eye disease, sarcoidosis, tear, Ferning Test, branching

## Abstract

Background: Sarcoidosis is a systemic inflammatory disease characterized by the formation of non-caseating granulomas, predominantly affecting the lungs and lymph nodes. However, the disease can affect any organ, including the eye, where it most commonly manifests as uveitis and dry eye disease (DED). The Ferning Test (FT), a non-invasive method for tear film analysis, offers insight into tear quality. Through this study, we aimed to evaluate the ability of the FT to diagnose and differentiate DED in patients with sarcoidosis. Methods: The study included a sample of 30 patients, divided into three groups, each consisting of 10 patients: one group of patients with sarcoidosis and dry eye disease (S-DED), one group of patients with DED without other systemic pathologies, and a control group of healthy individuals. Tear film samples were collected from the right eye of each participant, without stimulation, by microcapillarity. A drop of tear was spread on a microscope slide, allowed to dry, and then examined under a microscope to analyze the crystallization pattern. Results: Microscopic analysis revealed a significant difference in the structure and morphology of crystallization, as well as in the number of formed branchings, in sarcoidosis patients compared to patients in the other two groups. This finding suggests a distinct alteration in tear film composition in patients with sarcoidosis. Conclusions: Based on these results, the FT represents a valuable and promising tool for the diagnosis of DED associated with sarcoidosis. Being a non-invasive, easy-to-perform, and inexpensive test, it can be widely implemented in any ophthalmology department, opening perspectives for the test to become an important component among the diagnostic elements of dry eye syndrome in patients with sarcoidosis.

## 1. Introduction

Sarcoidosis is characterized by the appearance of non-caseating granulomas of unknown etiology [1]. Although it is a disease that can affect any organ, changes most often occur in the lung, followed by the lymph node system, skin, eyes, muscles, and brain [2]. It is more common in women aged 25–50 years, and ethnically, its prevalence is higher in African-American and Scandinavian populations [3]. The most common signs and symptoms include fever, weight loss, edema, cough, chest pain, ocular pain, and blurred vision [4].

Although it is an underdiagnosed pathology from the perspective of ophthalmological complications, studies in the specialized literature show that the prevalence of sarcoidosis is between 7 and 60% [1]. While anterior uveitis is the most common pathology, any other ocular structure can be affected (lacrimal gland, conjunctiva, optic nerve, retina, soft tissues, especially inferior orbital fat). Uveitis can cause significant ocular complications such as cataract, glaucoma, retinal vasculitis, vitreous opacities, and macular edema [5,6].

Dry eye disease (DED) is a multifactorial condition in which the homeostasis of the tear film is disrupted, leading to ocular discomfort, foreign body sensation, and a decrease in quality of life [7]. DED is a frequent comorbidity that appears as a component of involvement in sarcoidosis, and although it does not affect visual acuity like uveitis, it significantly impairs the quality of life in these patients, who often have other co-existing ailments related to the general context of the disease [5]. Ocular involvement in sarcoidosis frequently manifests before the diagnosis of the underlying systemic disease. Given the consequences of the pathology, the early diagnosis of the underlying disease is extremely important in patients presenting with ocular inflammation [3].

Ferning Test (FT) was introduced into clinical practice as an alternative to measuring tear osmolarity, the latter being extremely expensive due to the equipment used in tear analysis [8].

Even though the crystallization process of body fluids was described as early as 1791 by Fourcroy and Vauquelin, it was not until 1943 that Papanicolau observed the drying of cervical mucus in the form of ferns with preferential distributions [9].

Rolando, in 1984, introduced this test into ophthalmology, with the aim of diagnosing DED. Thus, in the original classification, desiccated tears are divided into 4 grades, depending on the presence or absence of arborizations. Grades 1 and 2 are normal, while grades 3 and 4 diagnose dry eye syndrome.

In 2013, Lopez Solis et al. individualized four distinct zones resulting from tear desiccation [10]. These zones included zone I, the transition band, zone II, and zone III. Later, Masmali et al. refined the classification process by introducing a five-grade scale, intended to eliminate the diagnostic inaccuracies for grades 1 and 2 associated with Rolando’s classification [11].

This article investigates tear film alterations in patients with sarcoidosis by employing the FT. The study introduces the use of FT in this context, establishing a novel approach to ocular surface assessment, with potential implications for improving diagnostic accuracy and enhancing the clinical understanding of ocular involvement in this systemic disease.

## 2. Materials and Methods

The study design and protocol were conducted in accordance with the tenets of the Declaration of Helsinki for research involving human subjects and approved by the Ethics Committee of “Grigore T. Popa” University of Medicine and Pharmacy Iasi, Romania (No. 446/approval date on 28 May 2024). Written informed consent was obtained prior to patient evaluation.

We analyzed a group of 10 patients known to have sarcoidosis and dry eye disease (S-DED). We enrolled another 20 patients, of whom 10 had DED without other pathologies, and another 10 healthy control patients. The inclusion criteria were patients with a confirmed diagnosis of S-DED confirmed by both pneumologist and ophthalmologist and patients with DED, defined by a positive Schirmer I test in the absence of concomitant ocular or systemic disease. Exclusion criteria comprised prior refractive or cataract surgery, refusal or inability to undergo ophthalmological assessment or to provide informed consent, and systemic medication use with potential effects on tear secretion.

The study protocol consisted of performing a Schirmer I test (without anesthetic) on the left eye. The technique involved placing a millimeter-numbered filter paper on the tarsal conjunctiva of the lower eyelid for 5 min, after which the wetting value of the paper was read. Based on these values, the 3 groups could be formed.

Concomitant with the sampling procedure, a volume of 1–3 μL was collected using a 10-μL micropipette tip in order to generate a greater vacuum effect, thereby facilitating a higher-quality tear collection, which was subsequently placed onto a glass slide. Tear collection by microcapillarity was performed on the right eye, so as not to interfere with the lacrimal stimulation effect induced by the Schirmer tests. This effect can modify tear composition by transforming tears with basal secretion into tears secreted as a result of stimulation. These were extracted from the outer canthus adjacent to the free margin to avoid lesions and corneal overstimulation. After a desiccation process lasting 10 min at a temperature of 25 degrees Celsius and ambient humidity of 47%, the samples were analyzed by phase-contrast microscopy using a Levenhuk MED D45T Digital Trinocular microscope (Levenhuk, Tampa, FL, USA). Photographs were taken with the main camera of an iPhone 15 Pro Max at a magnification of 1.6×, using a stand for the microscope eyepiece. Standardization of dimensions was performed by analyzing dried tears on a glass slide placed over the screen of an iPhone 15 Pro Max.

**Figure 1:**
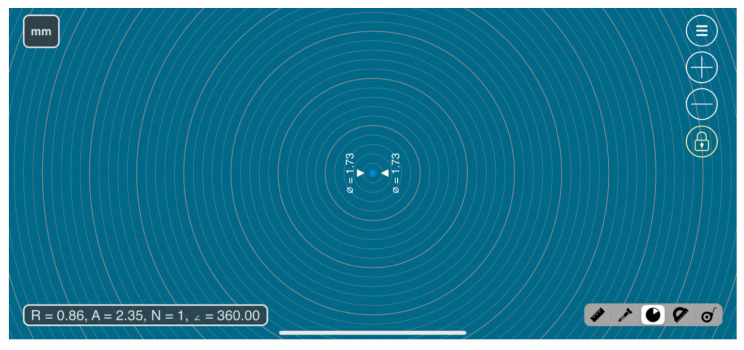
Image depicting the interface of the MilimeterPro application.

**Figure 2:**
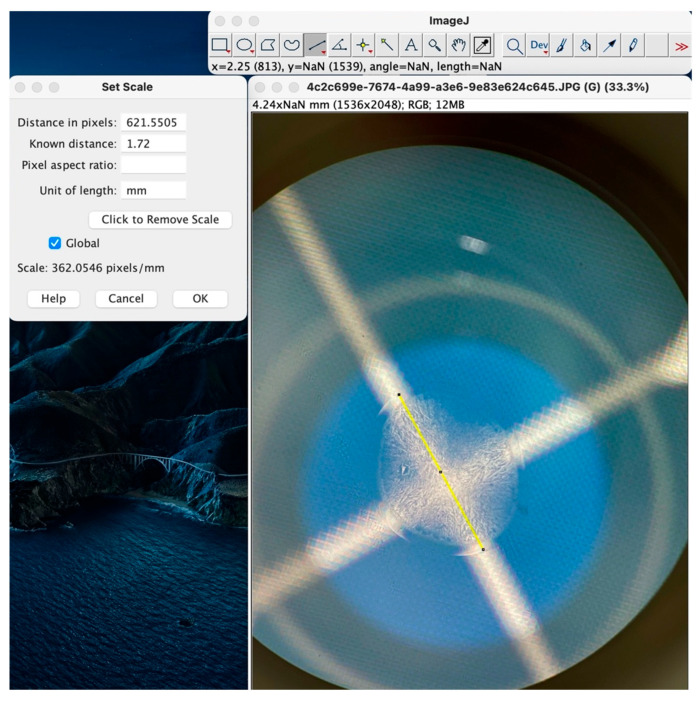
Standardization of tear dimensions—correlation between the microscopic image from the MilimeterPro application, the slide with the dried tear sample, and the ImageJ2 software.

Based on the MillimeterPro application, it was possible to calculate the area, diameter, and radius of the dried tears, which were then correlated with the pixel size of 50 mm with a diamond shape arrangement characteristic of these types of displays (Figure 1). Subsequently, these parameters were used to define the working scale in the ImageJ2 software (Figure 2).

The classification of DED and S-DED has been established based on the Schirmer I test, Rolando and the Masmali classification [10,11,12,13].

### Statistical Analysis

The data were loaded and processed using the statistical functions of SPSS 18.0, with a significance threshold set at 95%. A *p* value < 0.05 was considered statistically significant.

Data distribution was assessed using the Shapiro–Wilk test. Normality was confirmed for Schirmer test values, FT ramifications, and Brightness 205 and 155 across all groups. Regarding age, normality was respected in the DED and S-DED, but not in the control group. Between-group comparisons of FT, Brightness 205, and Brightness 155 values were performed using independent-samples t-tests. Equality of variances was verified with Levene’s test (*p* > 0.05).

Results are reported as mean ± standard deviation, unless otherwise specified. Outliers, defined as values more than 1.5 box-lengths from the edge of the box in boxplots, were examined and retained, as they were not considered extreme. Receiver operating characteristic (ROC) curve analysis was applied to Masmali, Schirmer, and Rolando scores to evaluate their diagnostic accuracy. Furthermore, a multivariate linear regression model was constructed with Brightness 205, Brightness 155, and FT values as dependent variables to investigate the contribution of clinical parameters to these outcomes.

## 3. Results

Following the analysis of the collected photographs, it was found that all patients diagnosed with sarcoidosis exhibited impairment of the tear film. Seven of these patients were classified as grade 3 according to Rolando/grade 4 according to Masmali, while three patients were classified as grade 2 Rolando/grade 3 Masmali.

Patients with sarcoidosis exhibited a total number of branches, quantified by ImageJ2, 4024 higher than that observed in healthy controls. Initially, the tears photographed post-drying were divided into four hourly quadrants, with the regions selected for analysis chosen randomly. The analysis excluded the central area of the tears because this region predominantly captures only zones 2 or 3, without including zone 1 and the transition band, which are essential for the emergence of the fern budding process.

The images were processed in ImageJ2 and standardized by selecting a brightness threshold of 155, representing the highest image parameter capable of accurately selecting the fern patterns and their branches. Starting from a brightness value of 155, increments of 5 were applied up to 205, which corresponds to the lowest image parameter capable of distinguishing the crystallization pattern. It was observed that these two endpoints of the interval were the most representative for variations in branch selection induced by the software. The images were subsequently processed through color threshold adjustment, binary transformation, and particle analysis.

**Figure 3:**
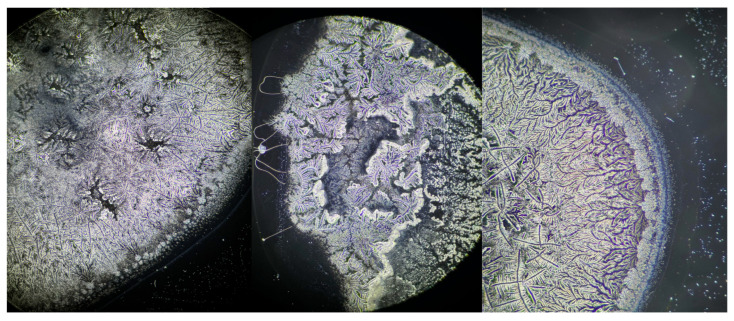
Appearance of fern patterns in a patient diagnosed with sarcoidosis (**left**) versus a control subject (**center**) versus a patient with DED (**right**).

**Figure 4:**
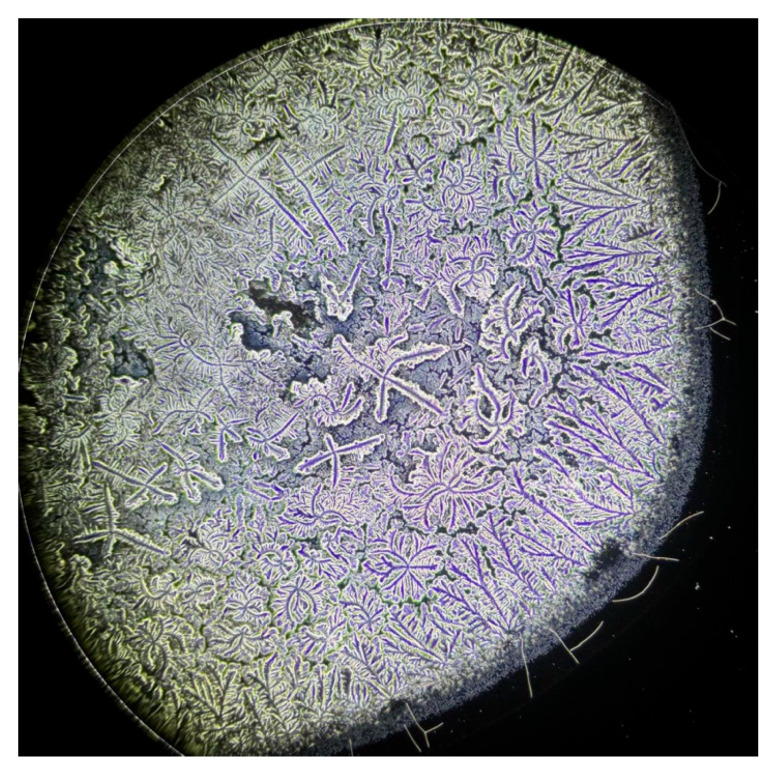
Dried tear sample evaluated by phase-contrast microscopy.

**Figure 5:**
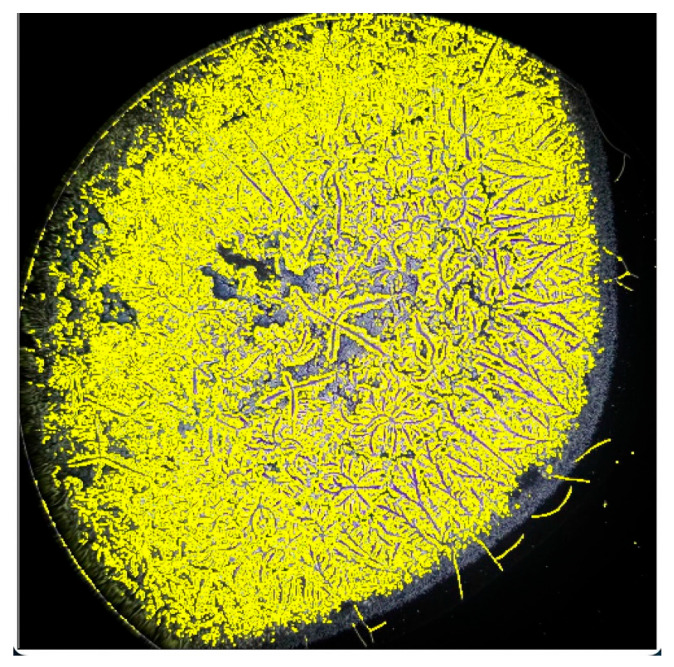
Selection of highlighted areas for conversion to a binary system.

**Figure 6:**
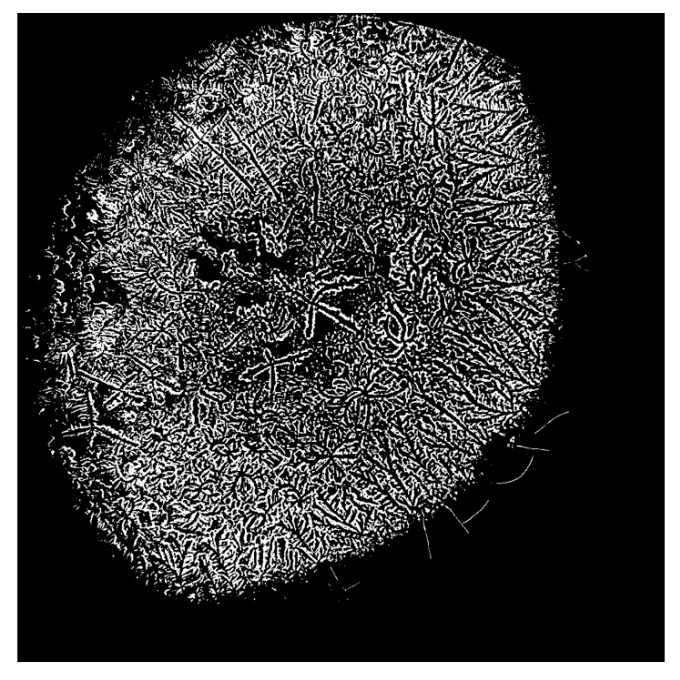
Binary system image of the dried tear sample.

**Figure 7:**
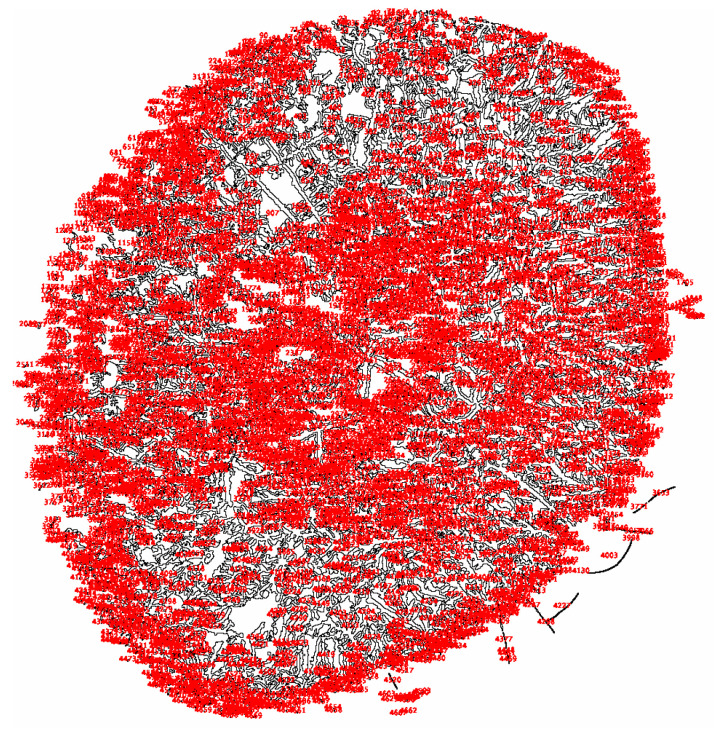
Number of branches quantified using ImageJ2.

This analytical method revealed the growth patterns of the ferns. In sarcoidosis patients, fern growth follows a linear pattern at a consistent height, whereas in healthy patients and those with DED, growth occurs at varying heights, fern over fern, complicating the quantification of branching structures (Figure 3). This observation is supported by attempts to convert the images into a binary system (Figure 4, Figure 5 and Figure 6). This constitutes the second reason for processing the analysis over a range of brightness values (Figure 7).

**Figure 8:**
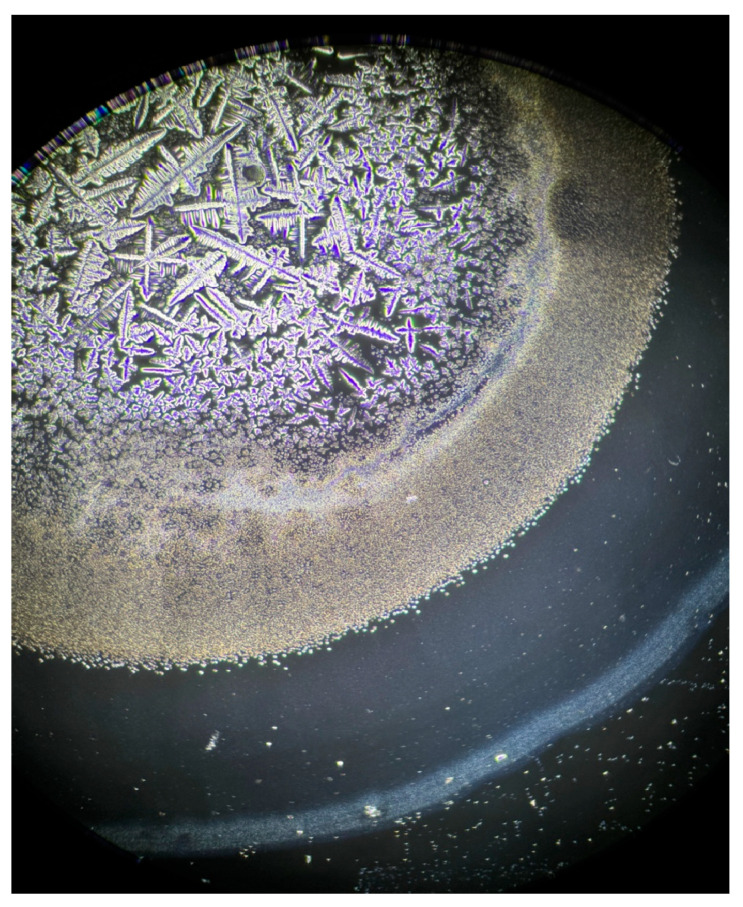
Appearance of dried tears in patients who received artificial tear treatment. Spherical granules are observed between layer I and the transition zone, likely serving a protective function.

**Figure 9:**
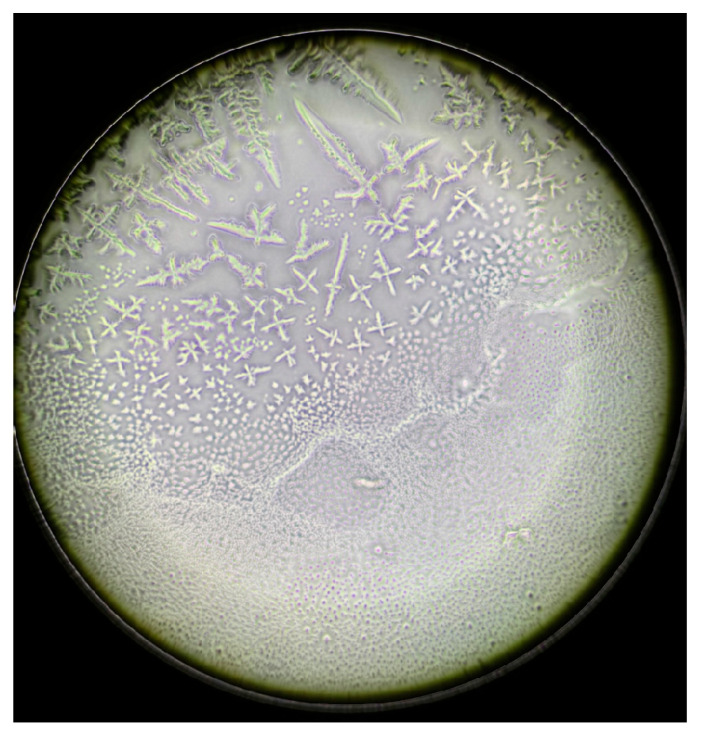
Magnified view of these spherical structures.

After the application of artificial tears (0.15% sodium hyaluronate), patients exhibited an improvement in grading by 1 to 2 levels. Furthermore, phase-contrast microscopy revealed a significant increase in layer I, accompanied by a transition zone densely saturated with small spherical formations resembling suspended lipids (Figure 8 and Figure 9). In vivo, these structures appear to serve as a protective barrier against desiccation.

The percentage distribution was homogeneous, with female patients predominating across all three study groups (*p* = 0.436) (Table 1).

Age group distribution showed a higher frequency of patients over 40 years old in both S-DED group and DED group (70% vs. 60%), whereas the control group was predominantly composed of patients under 40 years of age (90%; *p* = 0.01) (Table 1).

The mean level of the FT branching score was significantly higher in patients with S-DED compared to both the control group (6254.7 ± 1303.36; *p* < 0.0005) and DED group (5817.1 ± 1329.29; *p* < 0.0005). In contrast, the mean FT branching score did not differ significantly between the DED group and the control group (437.6 ± 812.8; *p* = 0.597) (Table 2).

Multivariate analysis using linear regression showed that FT branching values were not influenced by sex and/or age in either the S-DED group (adjusted R^2^ = 0.061; *p* = 0.668) or the DED group (adjusted R^2^ = 0.061; *p* = 0.668) (Table 3).

The mean level of standardized Brightness 155 was significantly higher in patients with S-DED compared to both the control group (3420.4 ± 1277.64; *p* = 0.015) and the DED group (4628.8 ± 1165.51; *p* = 0.001). In contrast, the mean standardized Brightness 155 level did not differ significantly between the DED group and the control group (1208.4 ± 816.75; *p* = 0.159) (Table 4).

Multivariate analysis using linear regression demonstrated that standardized Brightness 155 values were not influenced by sex and/or age in either the S-DED group (adjusted R^2^ = 0.236; *p* = 0.613) or the DED group (adjusted R^2^ = 0.035; *p* = 0.486) (Table 5).

The mean level of standardized Brightness 205 was significantly higher in patients with S-DED compared to both the control group (3963.7 ± 1498.39; *p* = 0.016) and DED group (4768.8 ± 1493; *p* = 0.005). In contrast, the mean standardized Brightness 205 level did not differ significantly between the DED group and the control group (8260 vs. 9065; *p* = 0.822) (Table 6).

Multivariate linear regression analysis showed that standardized Brightness 205 values were not influenced by sex and/or age in either the S-DED group (adjusted R^2^ = 0.251; *p* = 0.636) or DED group (adjusted R^2^ = 0.035; *p* = 0.486) (Table 7).

The data obtained from the application of the MASMALI and Rolando scoring systems, along with the Schirmer I test, highlight a significantly higher degree of ocular involvement in patients with S-DED compared to those with isolated DED and healthy controls. Specifically, 70% of patients in the S-DED group were classified as grade 2 according to the MASMALI score, indicating severe tear film dysfunction, whereas only 30% of DED patients and 20% of healthy subjects fell into the same category. Similarly, the Rolando score was pathological in 70% of S-DED patients, compared to 30% in the DED group and 20% in the control group. Regarding tear secretion, assessed via the Schirmer I test, only 30% of S-DED patients had normal values, compared to 10% in the DED group, while all participants (100%) in the control group showed normal results. These findings support the hypothesis that patients with sarcoidosis develop more severe ocular surface damage, with both structural and functional alterations of the tear film, distinct from those observed in isolated DED. This suggests a direct involvement of systemic inflammation in the pathogenesis of lacrimal gland dysfunction (Table 8).

**Figure 10:**
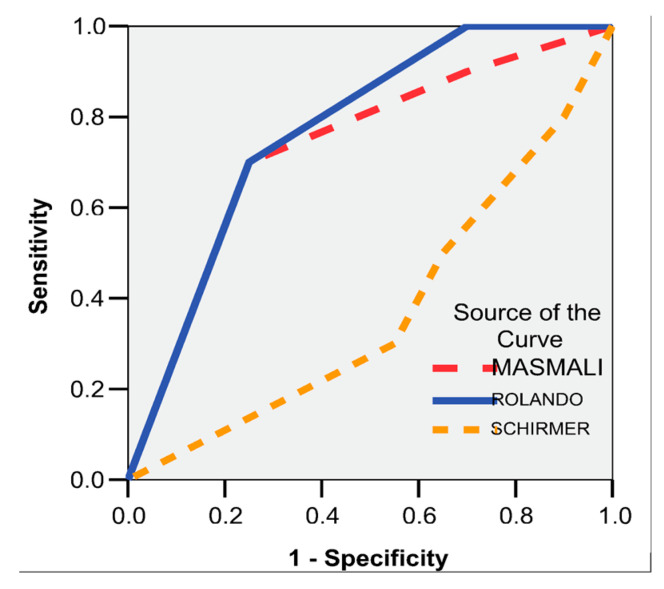
ROC curve. Dependent variable: S-DED. Independent variables: MASMALI, ROLANDO, SCHIRMER.

ROC curve analysis demonstrated that both the MASMALI and ROLANDO scores were valuable predictors of S-DED (Figure 10). The MASMALI score showed a sensitivity of 70% and a specificity of 75%, with an AUC of 0.733 (95%CI: 0.536–0.929; *p* = 0.041). Similarly, the ROLANDO score demonstrated a sensitivity of 71% and a specificity of 56%, with an AUC of 0.770 (95% CI: 0.599–0.941; *p* = 0.018).

**Figure 11:**
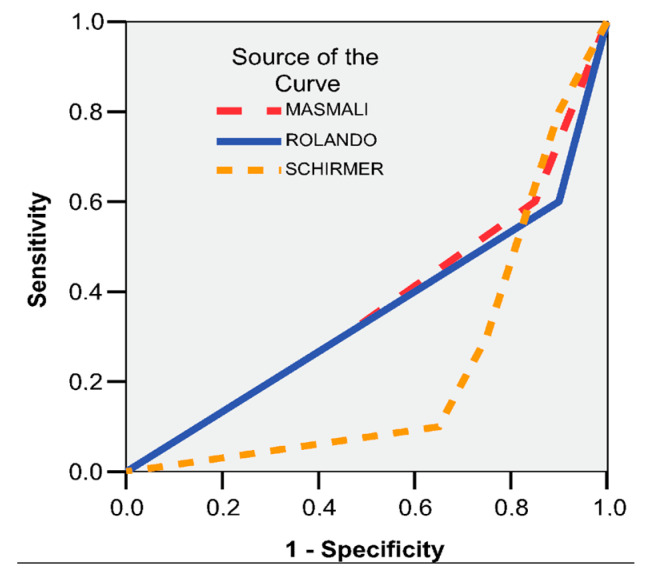
ROC curve. Dependent variable: DED cohort. Independent variables: MASMALI, ROLANDO, and SCHIRMER.

The diagnostic scores evaluated did not show adequate predictive value for DED, with all AUC values falling below 0.600 (Figure 11). The MASMALI score yielded an AUC of 0.368 (95% CI: 0.146–0.589; *p* = 0.244), while the ROLANDO score resulted in an AUC of 0.350 (95% CI: 0.126–0.574; *p* = 0.187). The SCHIRMER test demonstrated the lowest performance, with an AUC of 0.225 (95% CI: 0.051–0.399; *p* = 0.016), suggesting limited utility in the independent diagnosis of DED.

## 4. Discussion

The sex distribution analysis revealed a female predominance across all study cohorts (7 vs. 3 in S-DED 9 vs. 1 in DED, 7 vs. 3 in controls). This observation aligns with existing literature demonstrating that females exhibit a significantly higher susceptibility to developing DED [14,15]. Fluctuations in female sex hormones, particularly estrogen and progesterone, modulate lacrimal gland function and tear quality, contributing to this higher predisposition [16]. Correlating the findings with published data confirms that patients with sarcoidosis who develop DED are predominantly female. Furthermore, according to age distribution, most affected patients are women over 45–50 years old, consistent with previous study findings and observations reported in the scientific literature [17].

The mean branching level in the FT was observed to be noteworthy. This value was significantly elevated in the S-DED group compared to both the control group (6254.7 ± 1303.36; *p* < 0.0005) and the DED group (5817.1 ± 1329.29; *p* < 0.0005). Interestingly, no statistically significant difference in FT level was observed between the DED group and the control group (437.6 ± 812.8; *p* = 0.597). A distinctive qualitative observation supporting these results was the branching growth pattern: in S-DED, branches exhibited linear growth approximately at the same height, whereas in healthy subjects and DED patients, branches grew at varying heights with overlapping (one fern over another). This distinct distribution in S-DED not only facilitated branch quantification but may represent a key characteristic feature of the disease. Collectively, these data suggest that branch count could potentially serve as a specific biomarker for S-DED patients.

In existing literature, the FT and its quantification scores, such as those proposed by Rolando and Masmali, have already demonstrated diagnostic value in other pathologies. For instance, a landmark study by Norn reported that the Rolando score possesses an impressive sensitivity of 94% and specificity of 75% for diagnosing Sjögren’s syndrome [18]. Moreover, other studies confirmed the utility of this score in evaluating xerophthalmia and xerostomia associated with Sjögren’s syndrome [19], as well as in diagnosing keratoconjunctivitis sicca in rheumatoid arthritis patients [20], where it demonstrated high specificity. Considering these validations, the predictive value of FT score was specifically analyzed in S-DED. Analysis of the data indicated that both scores function as reliable predictors, with the MASMALI score demonstrating 70% sensitivity and 75% specificity, and the ROLANDO score exhibiting 71% sensitivity and 56% specificity. These findings underscore that the FT, together with the Masmali and Rolando systems, represents a promising diagnostic tool for screening and monitoring ocular involvement in sarcoidosis.

To ensure accuracy and avoid potential confounders, tear collection for the FT was performed without stimulation. This methodological choice is supported by previous studies demonstrating no statistically significant differences in tear crystallization test results regardless of whether the sample was collected with or without stimulation [21]. Additionally, non-stimulated collection was essential to avoid influencing Schirmer test values, which were performed concurrently in the contralateral eye, thus ensuring the integrity of both investigations’ data.

Although the exact mechanism of crystal formation in FT remains not fully elucidated, it is believed that the interaction between electrolytes, particularly sodium chloride (NaCl), and mucins plays a critical role in the morphology of ferning patterns [22,23,24,25]. In this context, it is noteworthy that in vernal keratoconjunctivitis, alterations in the mucous layer of the tear film are observed, potentially leading to abnormal Ferning patterns [26]. Within the present study, a distinct crystallization pattern was observed in patients with S-DED compared to those with DED without systemic pathology. This suggests that sarcoidosis patients might have a higher electrolyte concentration, such as NaCl, similar to what has been documented in Sjögren’s syndrome, where increased NaCl levels in the tear film have been observed [19,23].

Furthermore, the literature indicates an increased tear film evaporation rate in both Sjögren’s syndrome and sarcoidosis patients, which may contribute to tear hyperosmolarity [23,27]. A reduced tear film break-up time, frequently seen in these patients, likely results from structural changes in the lacrimal gland [28]. Morphological differences in Ferning crystals observed in patients with S-DED and Sjögren’s syndrome compared to those with isolated DED strongly support the notion that histological and biochemical alterations specific to these diseases confer a unique crystallization pattern [29,30,31]. This reinforces FT potential as a screening and differential diagnostic tool in managing patients with DED associated with systemic diseases. In this respect, evidence suggests that systemic diseases such as diabetes mellitus may predispose or accelerate DED development, with most diabetic patients exhibiting grade 2 DED according to Ferning patterns [32]. Smoking contributes to early onset and worsening of DED symptoms by disrupting the tear film, and obesity similarly impairs tear film stability, promoting the development of it [33,34]. Nonetheless, the unique ferning pattern results from complex interactions of multiple factors, requiring further studies for comprehensive understanding.

The application of artificial tears has demonstrated evident beneficial effects on FT morphology in DED patients, both in this study and others. Sodium hyaluronate 0.15% instillation was associated with a 1–2 grade improvement, accompanied by structural changes observable via phase-contrast microscopy, which may contribute to a protective effect against tear evaporation. These results align with international data reporting significant improvement in FT grading after artificial tear application in DED, but not in healthy subjects. Moreover, those studies highlight a progressive stabilization of the effect up to 180 min post-instillation. Thus, both sources support the notion that artificial tears not only temporarily hydrate the ocular surface but also contribute to rebalancing tear composition, directly impacting crystallization quality measurable by the FT [35].

Key limitations of the study include the relatively small sample size, the absence of hormonal assessment in female participants, and the lack of evaluation of systemic inflammatory markers in all participants, with no formal sample size calculation conducted. Nonetheless, this discovery may serve as a pivotal starting point, opening new directions for research and the exploration of diagnostic and monitoring methods in sarcoidosis. Future research should focus on larger patient cohorts, comprehensive assessment of hormonal and inflammatory markers, and a better evaluation of the therapeutic potential of artificial tears in normalizing DED parameters.

## 5. Conclusions

In conclusion, the FT demonstrates clinical relevance and significant statistical value in the assessment of DED in patients with sarcoidosis. Despite limitations, the findings, supported by the existing literature, highlight the considerable potential of this test. Both the Masmali and Rolando quantification scores, along with the distinctive morphological branching pattern, were identified as valuable predictive parameters, suggesting a possible unique tear film signature in sarcoidosis. Given its non-invasive, easy-to-perform, and cost-effective nature, the FT can be widely implemented regardless of medical center resources. Its broader use would facilitate the creation of extensive databases essential to further validate its diagnostic and monitoring relevance in DED. Moreover, such an approach may provide valuable insights into the underlying pathological mechanisms driving secondary DES development, opening new perspectives in research and management of systemic diseases.

## Figures and Tables

**Table 1:** Socio-demographic characteristics by study groups (n = 30).

Characteristics	S-DED Group (n = 10)			DED Group (n = 10)			Control Group (n = 10)			*p* *
	n	Mean ± SD	%	n	Mean ± SD	%	n	Mean ± SD	%	
Gender										0.436
Male	3		30.0	1		10.0	3		30.0	
Female	7		70.0	9		90.0	7		70.0	
Age										0.010
≤40 years	3		30.0	4		40.0	9		90.0	
>40 years	8		70.0	6		60.0	1		10.0	
Schirmer Value	14.4 ± 12.67			9.7 ± 7.09			24 ± 5.5			
Brightness 155	7618.8 ± 3408.59			2990 ± 1402.02			4198 ± 2169.12			
Brightness 205	13,028.8 ± 4189			8260 ± 2177.78			9065.1 ± 2214.48			
FT	11,704.3 ± 3745.07			5887.2 ± 1909.06			5449.6 ± 1721.01			

* Chi-square test. Likelihood Ratio.

**Table 2:** Data of FT branching scores by study groups.

Compared Groups	Mean Difference ± Std. Error Difference	*p*
S-DED vs. DED	5817.1 ± 1329.29	*p* < 0.0005
S-DED vs. Normal	6254.7 ± 1303.36	*p* < 0.0005
DED vs. Normal	437.6 ± 812.8	*p* = 0.597

**Table 3:** Multivariate analysis—Linear regression equation. Dependent variable: FT branching score. Independent variables: sex, age ^(a)^ *p* < 0.001, ^(ns)^ *p* > 0.05.

Group	N	Mean	Standard Deviation	Standard Error of Mean	Min	Max	t (Independent Samples Test)	*p*-Value
Total	30	7680	3849	702.6	3322	17,784	17.728	0.001
Control	10	5450	1721	544.2	3322	7950		
S-DED	10	11,704 ^(a)^	3745	1184.3	3791	17,784		
DED	10	5887 ^(ns)^ ^(a)^	1909	606.7	3529	10,533		

**Table 4:** Data of standardized Brightness 155 values by study groups.

Compared Groups	Mean Difference ± Std. Error Difference	*p*
S-DED vs. DED	4628.8 ± 1165.51	*p* = 0.001
S-DED vs. Normal	3420.4 ± 1277.64	*p* = 0.015
Normal vs. DED	1208.4 ± 816.75	*p* = 0.159

**Table 5:** Multivariate analysis—Linear regression equation. Dependent variable: Standardized Brightness 155. Independent variables: sex, age.

Group	Model	R	Adjusted R^2^	Std. Error	Coefficient B	t	*p*-Value
S-DED	Sex	0.020	0.125	0.001	272	0.104	0.920
	Sex, Age	0.197	0.236	0.039	35	0.530	0.613
DED	Sex	0.438	0.091	0.192	1611	1.061	0.324
	Sex, Age	0.500	0.035	0.058	35	0.736	0.486

**Table 6:** Data of standardized Brightness 205 values by study groups.

Compared Groups	Mean Difference ± Std. Error Difference	*p*
SAR vs. DED	4768.8 ± 1493	*p* = 0.005
SAR vs. Normal	3963.7 ± 1498.39	*p* = 0.016
Normal vs. DED	805.1 ± 982.17	*p* = 0.423

**Table 7:** Multivariate analysis—Linear regression. Dependent variable: Standardized Brightness 205. Independent variables: sex, age.

Group	Model	R	Adjusted R^2^	Std. Error	Coefficient B	t	*p*-Value
S-DED	Sex	0.630	0.321	0.397	2079	2.231	0.061
	Sex, Age	0.646	0.251	0.020	65	0.494	0.636
DED	Sex	0.438	0.091	0.192	1611	1.061	0.324
	Sex, Age	0.500	0.035	0.058	35	0.736	0.486

**Table 8:** The structure of the groups according to the Masmali, Rolando, and Schirmer I classifications.

Classification	S-DED Group (n = 10)		DED Group (n = 10)		Control Group (n = 10)		*p*-Value *
	n	%	n	%	n	%	
MASMALI							
Grade 0	0	0.0	4	40.0	2	20.0	0.036
Grade 1	3	30.0	3	30.0	6	60.0	
Grade 2	7	70.0	3	30.0	2	20.0	
ROLANDO							
Normal	3	30.0	7	70.0	8	80.0	0.040
Pathologic	7	70.0	3	30.0	2	20.0	
SCHIRMER							
Severe	2	20.0	2	20.0	0	0.0	0.001
Moderate	3	30.0	5	30.0	0	0.0	
Mild	2	20.0	2	20.0	0	0.0	
Normal	3	30.0	1	30.0	10	100	

* Chi-square test. Likelihood Ratio.

## Data Availability

The original contributions presented in this study are included in the article. Further inquiries can be directed to the corresponding author.

## Abbreviations

The following abbreviations are used in this manuscript:

DED	Dry Eye Disease
FT	Ferning Test
S-DED	Sarcoidosis and Dry Eye Disease
ROC	Receiver operating characteristic
AUC	Area under the curve
95%CI	Confidence Interval
NaCl	Sodium Chloride
